# First-line treatment options for advanced gastric/gastroesophageal junction cancer patients with PD-L1-positive: a systematic review and meta-analysis

**DOI:** 10.1097/MS9.0000000000000765

**Published:** 2023-05-03

**Authors:** Ling Fan, Ning Lu, Lingmin Zhang, Jie Zhang, Jie Li, Manli Cui, Mingxin Zhang

**Affiliations:** a Department of Gastroenterology, The First Affiliated Hospital of Xi’an Medical University; b Department of Anesthesiology, First Affiliated Hospital, Xi’an Jiaotong University, Xi’an; c Shaanxi University of Traditional Chinese Medicine, Xianyang, Shaanxi, China

**Keywords:** advanced GC/GEJC, Immune checkpoint inhibitors, PD-1 inhibitors, meta-analysis

## Abstract

Lately, many trials have paid much attention on the oncological outcomes of immunotherapy combined with chemotherapy as a first-line treatment. The authors perform a systematic meta-analysis to assess the efficacy and safety of programmed death 1 inhibitor plus chemotherapy for first-line treatment in advanced gastric/gastroesophageal junction cancer.

**Materials and methods:**

Literature search through major databases in English and Chinese: PubMed, Embase, Cochrane library, web of Science and CNKI updated on 10 March 2023. Randomized controlled trials were selected to investigate chemotherapy plus programmed death 1 inhibitor versus chemotherapy.

**Results:**

A total of 7 randomised controlled trials including 5788 participants were included. The overall survival (hazard ratio=0.79;95% CI: 0.74–0.85, *P*<0.01), progression-free survival (hazard ratio=0.72; 95% CI: 0.67–0.77, *P*<0.01) and objective response rate (risk ratio=1.24,95% CI: 1.18–1.31, *P*<0.05) were longer than chemotherapy alone in the pooled analysis. For subgroup analyses of overall survival, programmed death 1 inhibitors plus chemotherapy had a significant advantage in patients with combined positive score greater than or equal to 5, in Asia, in men and in those younger than 65 years (*P*<0.01), as were immune-mediated adverse events (odds ratio=8.86;95% CI: 1.26–62.47,*P*<0.05) and treatment-related grade 3–5 adverse events (odds ratio=1.40,95% CI:1.20–1.62, *P*<0.01).

**Conclusion:**

Programmed death 1 inhibitors plus chemotherapy have significant antitumour activity compared to chemotherapy alone. However, it is riskier in terms of toxicity than chemotherapy. The authors recommend programmed death 1 inhibitors plus chemotherapy as the optimal treatment regimen for patients with positive programmed death ligand 1 expression, in Asia, male and less than 65 years of age. More well-designed studies are needed to investigate the efficacy and safety of different immune plus chemotherapy drug doses and regimens.

## Introduction

Highlights

- Immunochemotherapy showed superior overall survival, progression-free survival and objective response rate.
- Immunochemotherapy had a risky toxicity profile as compared with chemotherapy.
- Immunochemotherapy is better for patients with combined positive score greater than or equal to 1, Asian and age younger than 65 years.

Gastric/gastroesophageal junction cancer (GC/GEJC) is a common malignant tumour in the upper gastrointestinal tract. It ranks as the fifth most common cancer and the fourth leading cause of cancer death worldwide^1,2^. In clinical practice, symptoms of early gastric cancer are often atypical, presenting as mild upper abdominal discomfort, resulting in more than 60% of patients having developed locally progressive disease by the time of initial diagnosis^3,4^. For first-line treatment of advanced metastatic HER2-negative gastric cancer, immune + chemotherapy regimens are recommended if combined positive score (CPS) greater than or equal to 5^5^. For first-line treatment of patients with advanced and metastatic HER-2-positive gastric cancer, trastuzumab in combination with ChT (capecitabine-oxaliplatin or 5-FU-oxaliplatin) is recommended^6,7^. Although chemotherapy regimens for progressive gastric cancer continue to be enriched, the number of patients with progressive gastric cancer has increased rather than decreased^6,8,9^. The 5-year survival rate for patients with advanced gastric cancer (stage IV) is only 6–14%^10^. Recently, immune checkpoint inhibitors (ICIs) have emerged as a treatment option for GC/GEJC. High mutational load and overexpression of immune checkpoint proteins characterise GC/GEJC, making treatment of patients with advanced GC/GEJC via the programmed death ligand 1 (PD-L1) pathway very promising^11–17^.

The programmed death 1 (PD-1) receptor is an immune checkpoint protein expressed on tumour cells and tumour-infiltrating immune cells that downregulates T-cell activation and evades immune responses to tumour cells^18^. When the immune checkpoint is suppressed, tumour cells subjected to T-cell attack may become more sensitive to cytotoxic drugs^19–22^. It has been shown that immunotherapy combined with chemotherapy can induce the host to produce durable and effective tumour antigen-specific T lymphocytes for a synergistic and optimised antitumour effect^23^.

However, the efficacy of immunotherapy in combination with chemotherapy for GC/GEJC is controversial. the results of the KEYNOTE-62 study in 2020 showed that PD-1 inhibitors in combination with chemotherapy were not significantly more effective than chemotherapy^24^. However, several recent clinical trials have shown the potential of PD-1 inhibitors in combination with chemotherapy in the first-line treatment of advanced gastric cancer^25–30^. In addition, the relationship between PD-L1 expression levels and efficacy and the safety of ICI in combination with chemotherapy still deserve further exploration^31,32^. Currently, no meta-analysis has explored the safety and efficacy of PD-1 inhibitors plus chemotherapy in first-line treatment for specific populations (age, sex, ethnicity and especially PD-1 positive expression) in advanced GC/GEJC. Therefore, we performed a meta-analysis of immunotherapy combination chemotherapy for patients with advanced G/GEJC and tried to determine the best population to benefit from immunotherapy in combination with chemotherapy.

## Methods

The review protocol was registered at PROSPERO (register number: CRD42022321710).The work has been reported in accordance with PRISMA (Preferred Reporting Items for Systematic Evaluation and Meta-Analysis) guidelines^33^. Self ^-^assessment of the quality of the systematic review using AMSTAR 2 criteria, with a very low AMSTAR 2 quality score^34^.

### Search strategy

Two investigators independently conducted a systematic search of the English-language literature published in PubMed, Embase, the Cochrane Library and Web of Science up to 10 March 2023, using the Mesh terms and the free keywords “anti-PD-1” or “anti-PD- L1” and “gastric cancer” and “cancer of the gastroesophageal junction”. We also searched both the American Society of Clinical Oncology (ASCO) and European Society of Medical Oncology (ESMO) meeting abstracts.

### Inclusion and exclusion criteria

Articles will be considered for inclusion if they meet the following criteria: (1) Participants: previously untreated, locally advanced, unresectable, or metastatic G/GEJC patients receiving first-line therapy. (2) Intervention: administration of chemotherapy plus PD-1/PD-L1 (3) Comparison: chemotherapy or placebo plus chemotherapy regimens. (4) Outcomes: studies containing information on one or all of the following: [overall survival (OS), progression-free survival (PFS), objective remission rate (ORR) and treatment-related adverse events (TrAEs)] (5) Study design: phase III randomised controlled trial. The following studies were excluded: (1) duplicate published data; (2) those with incomplete required information; (3) non-randomised controlled trials.

### Data selection

Two researchers independently extracted the extract contents from each trial. In case of disagreement, a third investigator helps resolve the disagreement or through discussion. We extracted the main categories based on the following: publication year, type, phase, treatment regimen, patient number and Asian.

### Statistical analysis

Statistical analyses were performed using Review Manager version 5.4 software (Revman; The Cochrane collaboration Oxford). The significance of heterogeneity was assessed using chi-square, and then the I² statistic was used to check the degree of heterogeneity across studies^3^. I² values greater than 50% indicated significant heterogeneity and a random effects model was used^35^. *P* value less than 0.05 was identified as statistically significant difference.

## Results

### Overview of literature search and study characteristics

**Figure 1:**
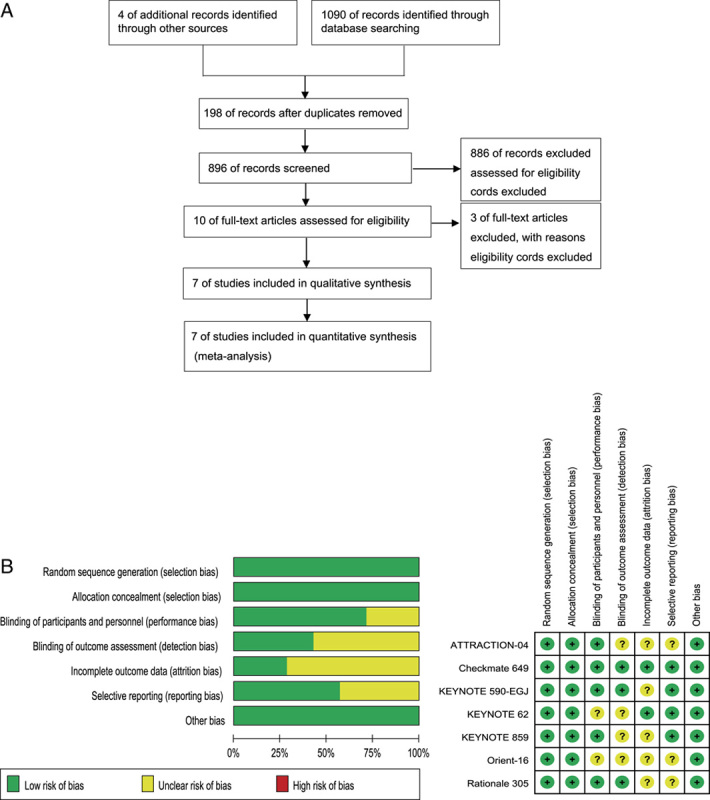
(A) Flowchart of the study selection process. (B) The risk of bias was evaluated by using the Review Manager 5.4. Risk of bias graph and risk of bias summary.

A total of 1094 records were retrieved. 198 records were excluded for duplicates, 886 records were excluded across exclusion criteria, 3 studies were excluded due to lack of the comparative data and 7 randomized clinical studies which compared the efficacy and safety of PD-1 inhibitor plus chemotherapy with placebo plus chemotherapy in advanced or metastatic GC/GEJC^24–30^. There were three trials of the KEYNOTE series that compared pembrolizumab plus chemotherapy with chemotherapy^24,25,29^. Two trials compared nivolumab plus chemotherapy with chemotherapy^26,28^. One trial comparing sintilimab plus chemotherapy versus chemotherapy^27^. One trial comparing tislelizumab plus chemotherapy versus chemotherapy^30^. The search process for this study was showed by flow diagram (Fig. 1 a). The main characteristics of the included trials were summarised in Table 1.

**Table 1:** General characteristics of the included literature.

	Type	Phase	Treatment regimen	No. Patient	Asian: %	Reference
KEYNOTE-062 (2020)	RCT	III	Pembrolizumab+ chemotherapy	257	24.9	^24^
			Chemotherapy	250	24.4	
KEYNOTE-590-GEJ (2021)	RCT	III	Pembrolizumab + chemotherapy	99	NA	^25^
			Placebo + chemotherapy	102	NA	
CheckMate 649 (2021)	RCT	III	Nivolumab + chemotherapy	789	16.4	^26^
			Chemotherapy	792	12.5	
ORIENT-16 (2021)	RCT	III	Sintilimab + chemotherapy	327	100	^27^
			Placebo + chemotherapy	323	100	
ATTRACTION-4 (2022)	RCT	III	Nivolumab + chemotherapy	362	100	^28^
			Placebo +chemotherapy	362	100	
KEYNOTE-859 (2023)	RCT	III	Pembrolizumab+ chemotherapy	790	33.3	^29^
			Placebo + chemotherapy	789	33.2	
Rationale 305 (2023)	RCT	III	Tislelizumab+ chemotherapy	274	73.7	^30^
			Placebo + chemotherapy	272	73.9	

RCT, randomized controlled trial; NA, not available.

### Risk-of-bias assessments

Risk of bias was assessed by two investigators separately. Study quality was validated using the Cochrane Collaboration’s “Risk of Bias” tool. The overall evaluation showed that most of the information was derived from studies with a low risk of bias (Fig. 1 b).

### Pooled analysis of OS comparing anti-PD-1 plus chemotherapy with chemotherapy

**Figure 2:**
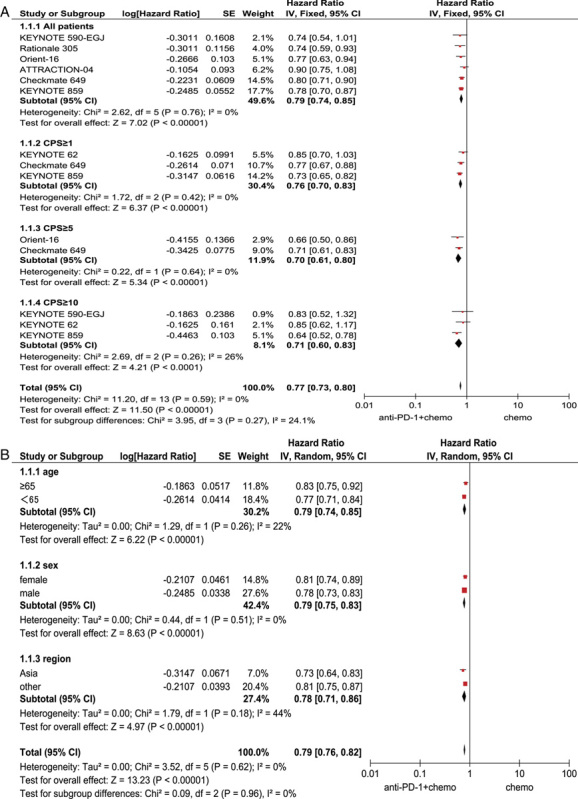
Pooled analysis of Overall Survival for anti-PD-1 plus chemotherapy compared to chemotherapy. (A) Pooled analysis forest plots showing overall survival for all randomised patients, CPS≥1, CPS≥5, CPS≥10. (B) Subgroup analysis forest plots illustrating the effect of age, sex and region on overall survival for all randomised patients. CPS, combined positive score; PD-1, programmed death 1; SE, standard error.

Pooling the OS demonstrated that the PD-1 inhibitor plus chemotherapy led to a 21% reduction in the risk of death compared with chemotherapy [hazard ratio (HR)=0.79; 95% CI: 0.74–0.85, *P*<0.01], and there was no obvious heterogeneity (I^2^=0%, *P*=0.76) (Fig. 2 a). Published data for PD-L1 expression subgroups were inconsistent across trials. To assess PD-L1 expression as a predictor of ICIs response, we pooled the available OS HRs for the chemoimmunotherapy subgroup according to the CPS of PD-L1. Patients with CPS greater than or equal to 1 (HR=0.76; 95% CI: 0.70–0.83, *P*<0.01) had better OS compared with all randomized patients (HR=0.79; 95% CI: 0.74–0.85, *P*<0.01). For those with CPS greater than or equal to 5, OS (HR=0.70; 95% CI: 0.61–0.80, *P*<0.01) had similar response to those with CPS greater than or equal to 10 (HR=0.71; 95% CI: 0.60–0.83, *P*<0.01). (Fig. 2 a).

In the comparison of the OS subgroup analysis, similar results were obtained to the overall population: the PD-1 inhibitor combined with chemotherapy was superior to chemotherapy alone. The pooled results showed that PD-1 inhibitors had significant benefits in the subgroups of Asian region, male and aged less than 65(Fig. 2 b).

### Pooled analysis of PFS comparing chemotherapy plus anti-PD-1 with chemotherapy

**Figure 3:**
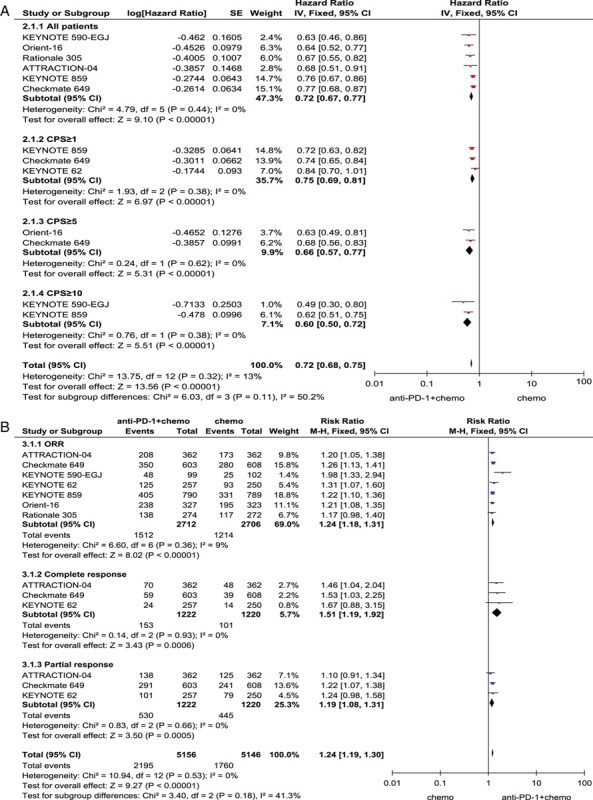
(A) Pooled analysis forest plots showing progression-free survival for all randomised patients, CPS≥1, CPS≥5, CPS≥10. (B) Pooled risk ratio (RR) for objective response rate (ORR), ORR subgroup (complete response vs. partial response) comparing anti-PD-1 plus chemotherapy with chemotherapy. CPS, combined positive score; PD-1, programmed death 1; SE, standard error.

Pooling the PFS showed that PD-1 inhibitor plus chemotherapy reduced the risk of disease progression compared with chemotherapy (HR=0.72; 95% CI: 0.67–0.77, *P*<0.01; and heterogeneity: I^2^=0%, *P*=0.44). Patients that over-expressed PD-L1 had better PFS. Patients with CPS greater than or equal to 10 (HR=0.60; 95% CI: 0.50–0.72, *P*<0.01) had the best PFS compared with patients with CPS greater than or equal to 5 (HR=0.66; 95% CI: 0.56–0.77, *P*<0.01) and CPS greater than or equal to 1 (HR=0.75; 95% CI: 0.69–0.81, *P*<0.01). CPS was positively correlated with PFS. (Fig. 3 a).

### Pooled analysis of ORR comparing anti-PD-1 plus chemotherapy with chemotherapy

The total number of objective responses reported in the literature for the PD-1 inhibitor plus chemotherapy treatment group was 2712, and the total number of objective responses for the chemotherapy treatment group was 2706.This meta-analysis showed that ORR was significantly improved in the treatment of the PD-1 inhibitor plus chemotherapy [risk ratio (RR)=1.24, 95% CI: 1.18–1.31,*P*<0.01；and heterogeneity:I^2^=9%, *P*=0.36]. In subgroup analysis, PD-1 inhibitor plus chemotherapy was superior to chemotherapy alone in both complete response (RR=1.51, 95% CI: 1.19–1.92, *P*<0.01; and heterogeneity: I^2^=0%, *P*=0.93) and partial response (RR=1.19, 95% CI: 1.08–1.31, *P*<0.01; and heterogeneity:I^2^=0%, *P*=0.66)(Fig. 3 b)

### TrAEs comparing chemotherapy plus anti-PD-1 with chemotherapy

**Figure 4:**
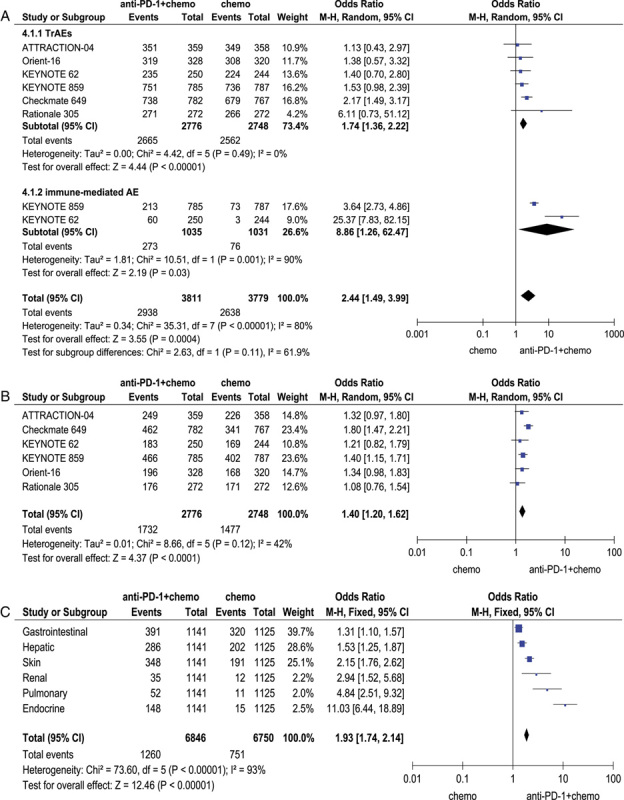
Pooled analysis of treatment-related adverse effects (TrAEs) Comparing chemotherapy plus anti-PD-1 with chemotherapy. (A) Pooled odds ratio (OR) for the incidence of all treatment-related adverse events and immune-mediated adverse events. (B) Pooled OR for the incidence of grade 3 or higher treatment-related adverse events. (C) Pooled OR for subgroups of the immune-mediated adverse events. PD-1, programmed death 1.

The pooling TrEAs [odds ratio (OR)=1.74, 95% CI: 1.36–2.22, *P*<0.01] and immune-mediated adverse events (OR =8.86, 95% CI: 1.26–62.47, *P*<0.01) exhibited a statistically significant difference (Fig. 4 a). Again, results showed that the difference of grade 3–5 serious adverse events between two groups was statistically significant (OR=1.40, 95% CI: 1.20–1.62,*P*<0.01) PD-1 inhibitors plus chemotherapy caused higher serious treatment-related adverse events (Fig. 4 b). Immune-mediated adverse events of the PD-1 inhibitor plus chemotherapy mainly involves endocrine, pulmonary and renal systems and etc. (Fig. 4 c).

## Discussion

Gastric cancer is one of the most common malignant tumours of the digestive system. Unfortunately, patients with advanced gastric cancer cannot be cured through surgery and have limited benefit from chemotherapy combined with targeted therapy. Immunotherapy, which has emerged in recent years, has been successfully applied to advanced head and neck squamous cell carcinoma^36–38^, non-small cell lung cancer ^39,40^, malignant melanoma^41–43^ and other solid cancers, and promising results have been achieved in studies of gastric cancer. Clinical trials of PD-1 inhibitors in combination with chemotherapy or as monotherapy for advanced gastric cancer are gradually underway^32,44^. However, the efficacy and safety of combination therapy using PD-1/PD-L1 inhibitors and chemotherapy in patients with advanced GC/GEJC remains controversial.Our meta-analysis investigated the efficacy and safety of anti-PD-1 with chemotherapy in the first-line treatment of advanced GC/GEJC, and to probe the differences in treatment efficacy among PD-L1 expression differences.

From this study, in terms of efficacy, PD-1 inhibitors combined with chemotherapy improved patients’ OS, PFS and ORR compared with chemotherapy treatment, and there was a statistical difference in the efficiency of the two treatment modalities. This result is consistent with the conclusion that chemoimmunotherapy is the best first-line treatment for HER2-negative, advanced gastroesophageal cancer, as concluded by the network meta-analysis of the Silva *et al.* in 2021^45^. In 2019, Chouaid *et al.* ^46^ conducted a study showing that the results of PD-L1 expression status plays an important role in predicting the efficacy of immune checkpoint inhibitors in predicting the efficacy of certain cancers. In 2020, the EPOC1706 trial results showed that the combination of immune and targeted therapy in PD-L1-positive patients got higher progression-free survival^47^. In our current subgroup analysis, we similarly found that CPS was positively correlated with PFS, with higher CPS being associated with higher PFS. For OS, patients with CPS greater than or equal to 10 (HR=0.71; 95% CI: 0.60–0.83, *P*<0.01)) had similar outcomes to those with CPS greater than or equal to 5 (HR=0.70; 95% CI: 0.61–0.80, *P*<0.01). the two subgroups of patients with high CPS expression responded better than patients with CPS greater than or equal to 1 (HR=0.76; 95% CI: 0.70–0.83, *P*<0.01) and all randomised patients (HR=0.79; 95% CI: 0.74–0.85, *P*<0.01) responded better. Therefore higher CPS scores were associated with a greater likelihood of better outcomes using immuno-combination chemotherapy.

In addition, our data suggest that patients in Asia, male patients and patients younger than 65 years of age may benefit from combination chemotherapy with ICIs. In 2015, Lin *et al.* ^48^ evaluated the gene expression profiles of more than 1000 gastric adenocarcinoma patients from Asian and non-Asian cohorts finding differential gene expression associated with immune function and inflammation in both populations. In this meta-analysis, significant differences were found between Asians (HR=0.73; 95% CI: 0.64–0.83, *P*<0.01) and non-Asians (HR=0.81; 95% CI: 0.75–0.87, *P*<0.01) in terms of improvement in OS, with results equivalent to previous studies. This suggests that ethnic differences in gastric cancer are an important stratification factor with possible implications for the efficacy of ICIs.

In this study we found that male patients (HR=0.78; 95% CI: 0.73–0.83, *P*<0.01) had a greater OS benefit with PD-1 inhibitors plus chemotherapy than female patients (HR=0.81; 95% CI: 0.74–0.89, *P*<0.01). A recent meta-analysis also showed that the relative benefit of immunotherapy in male cancer patients was greater than in female patients^49^. This finding also raises an important clinical question as to whether immunochemotherapy is more effective in men than in women in patients with advanced gastric cancer, which requires further investigation.

We also found greater OS benefit with PD-1 inhibitors plus chemotherapy in patients under 65 years of age (HR=0.77; 95% CI: 0.71–0.84, *P*<0.01). This is consistent with the results of a systematic review and meta-analysis published by Jiang *et al.* ^50^ in 2019 on cancer immunotherapy efficacy and patients’ age. This suggests that future studies should ensure greater inclusion of patients under 65 years of age in trials and focus on improving the effectiveness of immunotherapy in patients under 65 years of age.

However, in terms of safety, PD-1 combined with chemotherapy caused more treatment-related adverse events, and the drug toxicity of immune-combined chemotherapy could not be ignored. In this study, Immune-mediated adverse events of PD-1 inhibitor plus chemotherapy mainly involves endocrine, pulmonary and renal systems and etc. Combining several of the included studies, it appears that PD-1 inhibitor therapy caused the most common treatment-related adverse reactions included nausea, diarrhoea and fatigue. This is consistent with the results of a Meta-analysis published by Zhou *et al.* ^51^ in 2021 on treatment-related adverse events of PD-1 and PD-L1 inhibitor-based combination therapies in clinical trials. When these potential treatment risks are considered, it is beneficial to increase clinical vigilance, early recognition and intervention during the treatment process to prevent serious complications.

These TrAEs occur with ICI immunotherapy. They are referred to as “TrAEs of particular concern” or “immune-mediated adverse events.” Therefore, special attention should be paid to these adverse events. Our study provided modest evidence, but our study also has limitations. Given the lack of patient data and clinical heterogeneity between studies, the best option for the use of checkpoint inhibitors in the further treatment of patients with advanced GC/GEJC will be still debated. There is a great need for further high-quality studies, including additional data on different subtypes and larger randomized controlled trials, to confirm the efficacy and safety of alternative anti-PD-1/PD-L1 therapies for the treatment of patients with advanced GC/GEJC.

## Limitations

The use of immunotherapy in gastric cancer is in full swing, and there are similar systematic reviews of immune combination chemotherapy compared with chemotherapy for advanced gastric cancer^52,53^. However, no meta-analysis has yet explored the safety and efficacy of PD-1 inhibitors plus chemotherapy in the first-line treatment of specific populations (age, sex, ethnicity and especially PD-1 positive expression) in advanced GC/GEJC.Our study has several strengths:the rigorous methodology, the importance of the clinical question and the inclusion of a wide range of G/GEJC populations. As new clinical study data are reported, it is necessary to include new studies to improve the reliability of the paper. Although we have included the most recent clinical study data, the literature is still small and more clinical trials need to be included in the future to further improve the veracity of the paper’s conclusions. A high degree of heterogeneity in terms of disease indications could also not be excluded due to the lack of original patient and clinical data for inclusion in the literature.

## Conclusion

Our study shows that patients receiving PD-1 inhibitors in combination with chemotherapy have better survival endpoints in advanced GC/GEJC, especially for patients with positive PD-L1 expression, in Asia, in men and under 65 years of age. It also improves OS and PFS; however, the toxicities of immunotherapy in combination with chemotherapy cannot be ignored. From an efficacy point of view, immune checkpoint combination chemotherapy needs further trials to benefit patients with specific molecular subtypes and genomic alterations in a controlled and safe manner.

## Consent

None.

## Source of funding

This work was supported by Shaanxi Province Key Research and Development Program (2021SF-129 and 2022JM-502), Medical Project of Xi'an Science and Technology Bureau [2019114713YX002SF035(1)] and Youan Medical Alliance for the Liver and Infectious Diseases Research Special Fund (LM202028).

## Conflicts of interest disclosure

None.

## Presentation

None.

## Provenance and peer review

Not commissioned, externally peer-reviewed
